# A Manual of Auscultation and Percussion

**Published:** 1849-08

**Authors:** 


*A Manual of Auscultation and Percussion*; by M. Barth, agrege to the Faculty of Medicine, Paris, etc., and M. Henry Roger, Physician to the Bureau Central of the Parisian Hospitals, etc., etc. Translated, with additions, by Francis G. Smith, M. D., Lecturer on Physiology in the Philadelphia Association for Medical Instruction, one of the editors of the Medical Examiner, etc., etc. Second edition. Philadelphia: Lindsay & Blakiston, N. W. corner of Fourth and Chestnut streets; 1849, 18 mo. pp. 167.

This is an excellent little manual of the physical diagnosis of diseases of the chest and abdomen. The present edition is enriched by tables taken from the treatise of Dr. Walshe, modified to suit the text. The practitioner wll find it convenient as affording a *resume* of the more important principles and rules appertaining to the practice of Percussion and Auscultation. For sale by Derby & Co.

